# Type I Kounis Syndrome: Allergic Myocardial Infarction Triggered by Ciprofloxacin

**DOI:** 10.7759/cureus.62460

**Published:** 2024-06-16

**Authors:** Sangeetha Krishnamoorthy, Rashpinder Kaur, Nazeera Ameer, Amarachi A Uzoma, Nkechi R Enemuo, Shwetha Gopal, Vernice Glenn

**Affiliations:** 1 Internal Medicine, Trinity Health Oakland, Michigan, USA; 2 Microbiology, Kalpana Chawla Government Medical College, Karnal, IND; 3 General Practice, K S Hegde Medical Academy, Mangaluru, IND; 4 Family Medicine, Kharkiv National Medical University, Kharkiv, UKR; 5 Internal Medicine, College of Medicine, University of Ibadan, Ibadan, NGA; 6 Cardiovascular Disease, Bassett Medical Center, Cooperstown, USA; 7 Internal Medicine, Saint James School of Medicine, Arnos Vale, VCT

**Keywords:** drug allergy, fluoroquinolones, ciprofloxacin, kounis syndrome, allerigc myocardial infarction

## Abstract

Kounis syndrome (KS), known as allergic myocardial infarction (MI), is an uncommon but potentially life-threatening disease characterized by acute coronary artery disease (CAD) in the setting of allergic reactions. KS is most frequently triggered by medication, and ciprofloxacin-induced KS-I is rarely reported. Here, we present a case of KS-I triggered by ciprofloxacin in a young female with no prior CAD. A 35-year-old female presented with sudden onset chest pain, diaphoresis, and lightheadedness, accompanied by itching, confusion, and collapse, shortly after taking oral ciprofloxacin. Her electrocardiogram showed inferior wall MI with elevated cardiac troponin levels. Urgent coronary angiography was unremarkable. Her condition improved after sublingual nitroglycerine, methylprednisolone, and intramuscular injection of epinephrine. This case highlights the importance of recognizing drug-induced allergic reactions as a potential cause of acute coronary events, particularly in young patients without traditional risk factors.

## Introduction

Kounis syndrome (KS), an allergic myocardial infarction (MI), is a rare but potentially life-threatening condition characterized by the co-occurrence of acute coronary syndromes with allergic, hypersensitivity-related, or anaphylactic reactions in response to activation and degranulation of mast cells and release of histamine [1]. Coronary vasospasm, acute MI, or stent thrombosis can be the manifestation of KS. Triggering events of KS may involve medications, foods, certain conditions, and environmental exposure [2]. Among drugs, penicillin and cephalosporin are the most common culprits [3]. Ciprofloxacin-induced KS is rare and not widely reported in the literature. Only a few cases of KS triggered by ciprofloxacin have been underlined [4]. We report a case of ciprofloxacin-induced allergic MI (KS) in a young female.

## Case presentation

A 35-year-old female was brought to the emergency department with sudden onset severe chest pain and mild dyspnea followed by loss of consciousness and collapse. Her symptoms were preceded by chest tightness, sweating, nausea, dizziness, itching and rash on her lower limb, which began shortly 15 minutes after ingestion of ciprofloxacin 500 mg, prescribed by her physician to treat uncomplicated urinary tract infection. She had no previous medical history or family history of any disease. She was not allergic to any drug and had no history of travel, trauma, alcohol abuse or illicit drug use.

**Figure 1:**
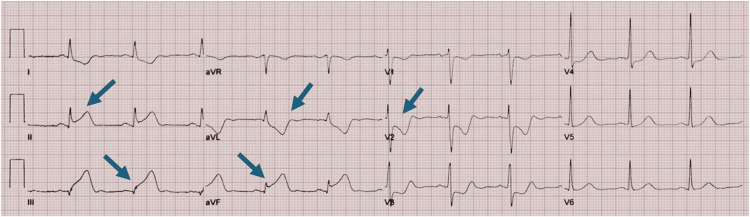
ECG showing ST-segment elevation leads II, III, aVF with ST-depression in reciprocal leads (blue arrows).

On evaluation, she looked anxious and diaphoretic but was well-oriented to time, place, and person. Her blood pressure was 80/50 mmHg, heart rate was 57/minute, and oxygen saturation was 85% on room air. Her respiratory and cardiovascular examinations were normal. She was immediately managed with intravenous methylprednisolone (40 mg), dexamethasone (5 mg), epinephrine (0.5 mg), and hydroxyzine (150 mg). Her electrocardiogram (ECG) revealed ST segment elevation in leads II, III, and aVF with ST-segment depression in the reciprocal leads (Figure 1). Her initial laboratory evaluations are shown in Table 1.

**Table 1:** Results of initial laboratory evaluations. CK: creatine kinase; MB: myocardial band; PaCO_2_: partial pressure of carbon dioxide; PaO_2_: partial pressure of oxygen; pH: potential hydrogen.

Parameter	Lab value	Reference value
White cell count	8200	4000-11000/mm^3^
Hemoglobin	12.5	12-16 g/dl
Platelet cell count	225,000	150,000-350,000/mcl
Serum creatinine	0.9	0.8-1.3 mg/dl
Blood urea nitrogen	18	08-25 mg/dl
Cardiac troponin	5	0-30 pg/ml
Creatine kinase	6.2	0-3.7 ng/ml
CK-MB	11	< 5 ng/ml
Arterial blood gas analysis		
PaCO_2_	40	35-45 mmHg
PaO_2_	65	75-100 mmHg
Serum lactate	1.9	0-2 mmol/l
pH	7.36	7.35-7.45

**Figure 2:**
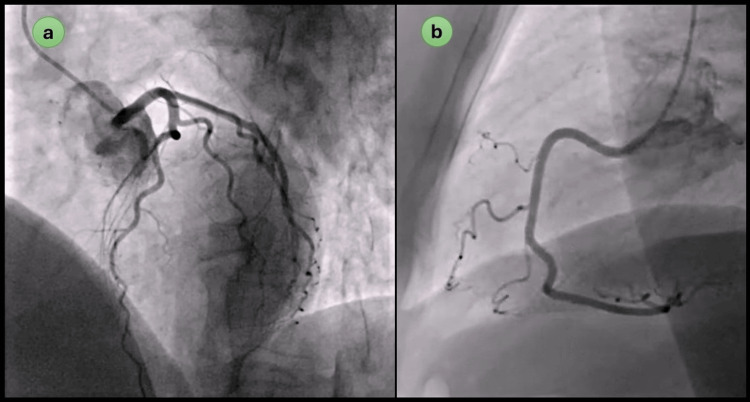
Coronary angiography revealing no significant stenosis or thrombosis in left (a) and right (b) coronary arteries.

A probable diagnosis of acute MI was made, and she was managed with supplemental oxygen, oral aspirin (300 mg), oral clopidogrel (180 mg), and injection heparin (3000 units) and became hemodynamically stable. She was taken to the cath lab, and her angiography revealed no significant stenosis or thrombus in the coronary arteries with thrombolysis in MI (TIMI) blood flow was observed to grade III (Figure 2). The echocardiogram showed no segmental motion wall abnormality with an ejection fraction of 60%.

**Figure 3:**
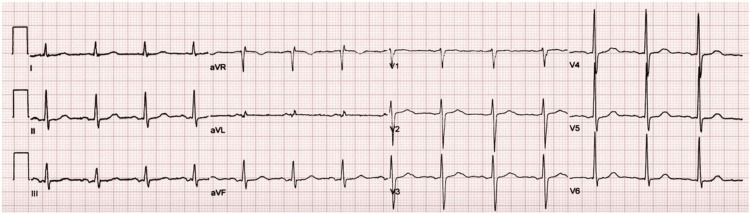
ECG with no significant changes.

Based on her clinical symptoms, laboratory findings, and angiography findings, a probable diagnosis of allergic MI (KS-I) was made. The next day, her repeat ECG showed no abnormality, and the bedside echocardiogram revealed a normal ejection fraction. Her condition improved gradually, and she was discharged on day seven (Figure 3). On follow-up, she was hemodynamically stable with no changes on ECG and electrocardiogram.

## Discussion

**Figure 4:**
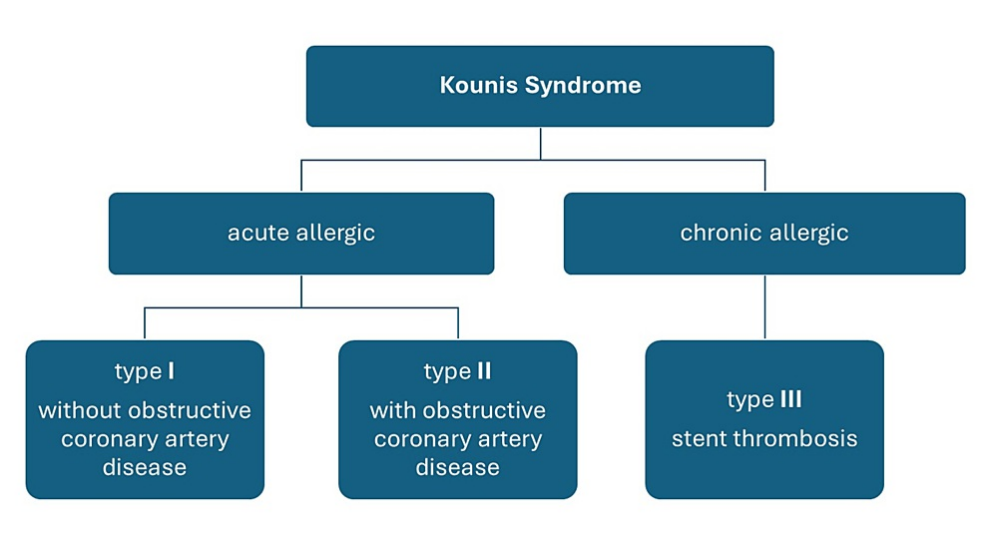
Kounis syndrome and its types. Reference: [6]

The association of cardiovascular manifestations and hypersensitivity reactions has been well-established since 1991 when the first case of KS was reported as MI or coronary vasospasm could be triggered by a severe allergic reaction in the setting of mast cell degranulation and histamine release [5]. Due to severe morbidity and mortality, KS requires urgent evaluation and appropriate management. KS has been classified into three subtypes, as shown in Figure 4 [6]. KS is an uncommon condition with extremely low annual incidence (0.02%) and all allergic individual admissions (3.4%) [7]. KS can occur at any age with no genetic predisposition. Diabetes, hypertension, smoking, hyperlipidemia, and previous history of allergic reactions are among the risk factors [7].

Due to the rarity of the disease, only a few cases of ciprofloxacin-induced KS have been published. Navarro-Navajas et al. reported a case of ciprofloxacin-induced KS in a 71-year-old male. His symptoms began after ciprofloxacin ingestion for urogenital infections. ECG was suggestive of acute MI. However, coronary angiography confirmed the diagnosis of KS provoked by ciprofloxacin after ruling out all other possible causes [8]. Almedia et al. also published a case of ciprofloxacin-induced KS in an 85-year-old male who was administered ciprofloxacin as prophylaxis during an elective surgical procedure. He developed symptoms, and ECG was suggestive of acute coronary syndrome. Coronary angiography excluded coronary artery disease. His symptoms resolved after withdrawal of the drug [9].

**Figure 5:**
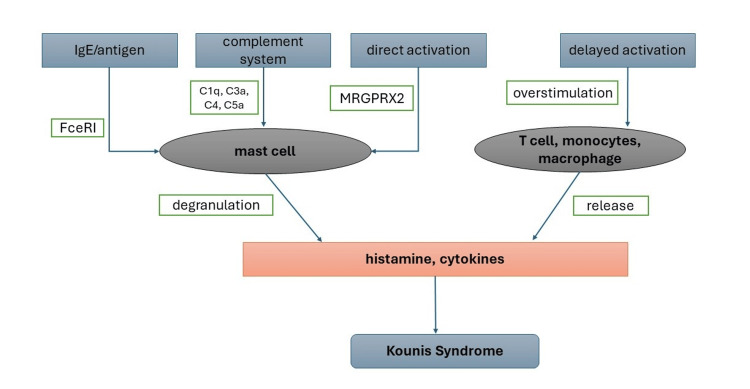
Pathophysiology of Kounis syndrome. IgE: immunoglobulin E; FceRI: Fc region of immunoglobulin E; MRGPRX2: mas-related G-protein coupled receptor member X2; C: complement Reference: [10,11]

**Figure 6:**
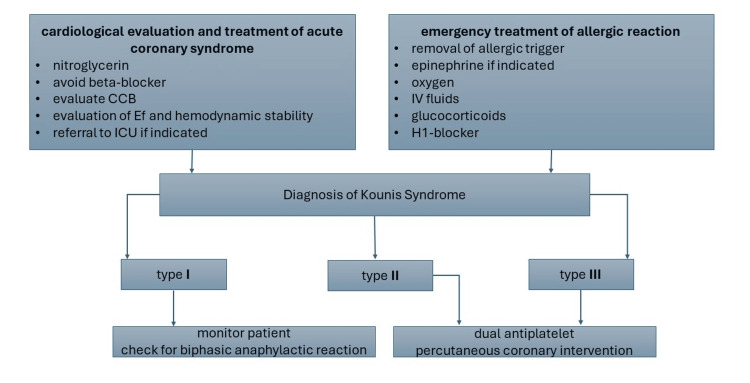
Approach to Kounis syndrome management. CCB: calcium channel blocker; IV: intravenous; ICU: intensive care unit; H: histamine [12]

Pathophysiology of KS involves activation and degranulation of inflammatory mediators, including mast cells, in the setting of allergen crosslinking to immunoglobulin E antibodies attached to the surface of mast cells [10]. Degranulation of mast cells releases histamine and other cytokines, including chemokines, tryptase, and arachidonic acid, which cause coronary vasoconstriction, platelet activation, and intimal thickening. The release of histamine and proteases contributes to plaque erosion and rupture. Arachidonic acid and thromboxane products induced platelet aggregation. The combined effect of these mediators is a pro-thrombotic environment leading to coronary spasm or thrombosis (Figure 5) [10,11]. Managing KS is challenging due to conflicting approaches to cardiovascular and allergic reaction treatments, as shown in Figure 6 [12]. Management of KS involves removing the offending allergen, managing the acute coronary vasospasm, and treating the allergic response. Careful selection and use of medicines are needed when managing the acute condition to avoid further histamine release or exacerbation of coronary vasospasm.

## Conclusions

Although rare, type I KS is a life-threatening adverse event of drug-induced allergic reactions. KS is challenging to diagnose due to mixed clinical presentations of acute coronary syndrome and allergic reactions. KS should be included in the differential diagnosis of patients presenting with acute coronary syndrome without typical risk factors after drug-induced allergic reactions, especially ciprofloxacin. Early recognition and appropriate management are crucial for optimum outcomes.

## References

[1] Kounis NG (2013). Coronary hypersensitivity disorder: the Kounis syndrome. Clin Ther.

[2] Kadeli D, Mangesh D, Keshava R, Gopi A (2019). Kounis syndrome: allergic myocardial infarction!!. J Indian Coll Cardiol.

[3] Sunder A, Mohanty B, Singh A (2020). Kounis syndrome: a rare case. J Family Med Prim Care.

[4] Koniari I, Kounis NG, Soufras G, Tsigkas G, Hahalis G (2017). Quinolone-induced hypersensitivity reactions and the Kounis syndrome. Rev Port Cardiol.

[5] Kounis NG, Zavras GM (1991). Histamine-induced coronary artery spasm: the concept of allergic angina. Br J Clin Pract.

[6] Kounis NG, Mazarakis A, Tsigkas G, Giannopoulos S, Goudevenos J (2011). Kounis syndrome: a new twist on an old disease. Future Cardiol.

[7] Li J, Zheng J, Zhou Y, Liu X, Peng W (2018). Acute coronary syndrome secondary to allergic coronary vasospasm (Kounis Syndrome): a case series, follow-up and literature review. BMC Cardiovasc Disord.

[8] Navarro-Navajas A, Casallas I, Isaza D, Ortiz P, Baracaldo-Santamaría D, Calderon-Ospina CA (2022). Type III Kounis syndrome secondary to ciprofloxacin-induced hypersensitivity. Medicina (Kaunas).

[9] Almeida J, Ferreira S, Malheiro J (2016). A rare cause of acute coronary syndrome: Kounis syndrome. Rev Port Cardiol.

[10] Alblaihed L, Huis In 't Veld MA (2022). Allergic acute coronary syndrome-Kounis syndrome. Emerg Med Clin North Am.

[11] Ou W, Wang B, Zhang G (2023). Acute myocardial infarction after inactivated COVID-19 vaccination: a case report and literature review. Front Cardiovasc Med.

[12] Fassio F, Losappio L, Antolin-Amerigo D (2016). Kounis syndrome: a concise review with focus on management. Eur J Intern Med.

